# Possibilities of Checking Water Content in Porous Geopolymer Materials Using Impedance Spectroscopy Methods

**DOI:** 10.3390/ma16145190

**Published:** 2023-07-24

**Authors:** Dariusz Mierzwiński, Janusz Walter, Dominika Wanat

**Affiliations:** Faculty of Materials Engineering and Physics, Cracow University of Technology, 24 Warszawska Street, 31-155 Kraków, Poland; dariusz.mierzwinski@pk.edu.pl (D.M.); dominika.wanat@student.pk.edu.pl (D.W.)

**Keywords:** geopolymer, porous material, impedance measurements, Nyquist plots, Bode plots, porosity

## Abstract

The porous geopolymer has been tested for its content of water using impedance methods. The pores of the material were filled with distilled water using a desiccator and a vacuum pump. An analysis of differential scanning calorimetry (DSC) was carried out in the next step to check the content of water, porosity and approximate value of specific heat of the geopolymer. Additionally, mercury porosimeter has been used for checking the porosity. The geopolymer material characterized in this way was subjected to impedance tests aimed at developing a quick method for assessing the water content in the material. Impedance measurements have been realized on an electrochemical workstation applying a 50 mV non-destructive amplitude of the potential and a frequency range of 1 Hz to 100 kHz. Change in the module of impedance and the phase shift angle were measured while the material was dried out. Significant differences were observed. The obtained graphs were simulated using a schematic model consisting of constant phase elements (CPEs) and a resistor (R). These values showed mechanisms of charge conduction. A simple method for assessing the water content of a porous geopolymer has been proposed in this paper. The real and imaginary impedance values were shown in Nyquist graphs. These graphs have characteristic maxima that move according to a linear equation with decreasing water content. Changes in Nyqiust charts are clearly visible even with small changes in the water content of the material and can be very useful for assessing it.

## 1. Introduction

Recently, we have seen widespread interest in pro-ecological solutions that aim to reduce global warming and pollution generated by human activity. One of the solutions to these problems is an attempt to reduce the production of ordinary Portland cement, during which a huge amount of greenhouse gases, including CO_2_, is emitted into the atmosphere. Geopolymers are a pro-ecological alternative to Portland cement concrete. Geopolymers are a group of cement aluminosilicate binders with diverse and adjustable material properties [1,2]. They are synthesized by adding an alkaline solution, called an activator, that most often consists of sodium hydroxide NaOH and sodium silicate Na_2_SiO_3_, which are the sources of sodium and silicon. Examples of such materials are blast furnace slag, metakaolin or fly ash, which is a by-product of coal combustion [3]. The main advantage of these materials is their manufacturing process, which can take place in a relatively low temperature starting from ambient temperature to about 80 °C [4,5,6,7,8,9]. They have a lower carbon footprint than traditional Portland cement, making them a more attractive alternative for sustainable construction [10,11,12,13].

Moreover, geopolymers are characterized by hardness and mechanical resistance comparable to ceramics and have very good resistance to aggressive chemical environments. Geopolymer is a porous material with many excellent properties such as high compressive strength, good electrical insulation and resistance to chemical corrosion. Furthermore, the widespread pores in geopolymer matrix provide enough channels for ion storage and motion [8,14,15,16,17,18]. The interests of researchers in the world oscillate around alkali-activated materials, which include geopolymers. Mechanical properties are affected by many factors, such as porosity, concentration of alkaline solution, and particle size of materials. These properties can also be changed by the additives used, such as glass fiber, glass waste, or natural fibers or other waste materials [19,20,21]

These are materials the porosity of which depends, among others, on the concentration of the alkaline activator used [6,8,22,23,24,25]. The methods of testing this geopolymer property are very important, especially when they are used in an environment with high humidity or catalyst matrix. Porous geopolymers stored in a humid environment absorb water, the presence of which could promote the growth of algae and fungus and decrease its properties.

The geopolymer is a heterogeneous mixture of materials with an internal pores. Depending on the degree of saturation of the pores with water, the concrete will have different conductive properties. Concrete may have a high electrical conduction when it is wet, but the same concrete will have a much lower conduction when it is dry.

The measurement of electrical conduction seems to be a simple measurement, but the physical and chemical properties of concrete make the use of direct current for such a measurement impossible. Because direct current techniques are not suitable for measuring electrical resistivity, alternating current should be used for measurements. Ceramic materials such as geopolymers are essentially non-conductive and therefore all observed conduction is due to the movement of ions in a network of interconnected pores. The high conduction can partly be represented by resistance, but as the number of conductive ions decreases due to water evaporation, conduction becomes more capacitive. First, the large pores are dried and then the smaller and deeper ones, which significantly change the conduction mechanism. These mechanisms also depend on applied frequency because ions move back and forth as a result of the changing direction of the voltage forcing them to move. Sinusoidal changing of voltage can have a very small value so it does not cause any changes in the cement. 

We applied a method based on impedance measurements using changing frequency and current, known as EIS (electrochemical impedance spectroscopy) [26,27,28,29,30,31,32,33].

Electrical conduction of porosity materials has been investigated extensively in the past, including EIS measurements [5,26,28,29,30,31,32,34,35,36,37,38,39,40,41]. In our work, we focused on applying impedance spectroscopy investigations to check water amounts in cements. A specially prepared system of stainless steel electrodes embedded in cement was built to improve the electrical connection between the geopolymer and the electrodes. The conduction between the electrodes was investigated using impedance methods and analyzed, among others, using Bode and Nyquist plots. An in-depth analysis of the results indicated the possibility of performing indicative measurements using a regular frequency generator. The measurement of water content in a geopolymer, which by its nature is a porous material, can be of significant importance during the production of geopolymers and their exploitation.

## 2. Materials and Methods

### 2.1. Geopolymer Preparation

Fly ash (FA) is one of the basic raw materials used in the production of geopolymer materials and is divided into three types according to ASTM 618-03. Our research was carried out using fly ash class F. Its chemical composition is given in Table 1.

**Figure 1:**
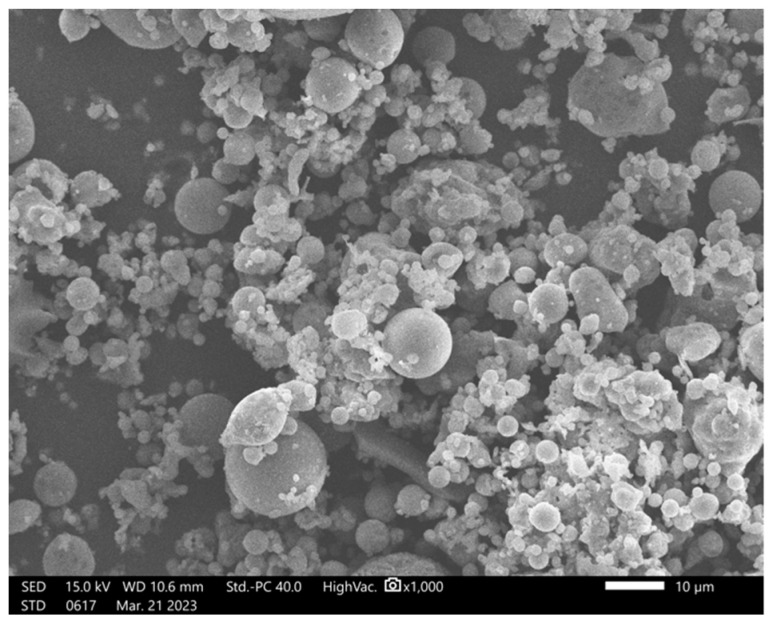
SEM fly ash morphology.

In our research, we used FA from the CHP plant in Skawina (Poland), whose micro-photograph is shown in Figure 1.

The oxygen composition of the tested ash and its properties are shown in Table 2 and Table 3. CEN reference sand was added to the geopolymer composition as a fine aggregate. in a 1:1 ratio (FA to sand). First, the dry mass was mixed to obtain a homogeneous mixture. Then, the previously prepared alkaline activator solution was added to the prepared mass in an amount sufficient to obtain a semi-liquid mass that could fill the mold. The alkaline activator used in this study comprises a sodium hydroxide solution containing sodium silicate additives. The solution was prepared by mixing volumes of NaOH = 7.4%, H_2_O = 10.9% and sodium water glass = 71.6%. The prepared mixture of the solid particles and the solution of the activator were homogenized for about 5 min. After preparing the geopolymer mortar, it was used to cast the mold with electrodes. The material was placed in the dryer set at 70 °C to avoid the formation of micro-cracks that could occur at a higher temperature closer to the boiling point of water [20].

After solidification, the obtained geopolymer was washed by immersing and holding it in distilled water to remove residues of the activator and other soluble substances. Immersion of the material in distilled water increases its conduction, which is a result of leaching of substances soluble in water. The water was periodically replaced until its conduction stabilized at a level indicating that the leaching has stopped [42]. Next, the geopolymer was totally dried using a laboratory dryer at a temperature of 70 °C for 80 h. Impedance measurements of the completely water-free material were carried out in the next step. 

Preparations of the geopolymer for impedance tests started with using water to fill the pores.

Pores were filled with distilled water using desiccator and vacuum pump. The water was previously de-aerated in vacuum and used as a medium in which the material was immersed. Starting the pump caused the material to be degassed and its pores to be filled with water. This process takes two days.

The content of water was checked in the following step via differential scanning calorimetry (DSC) analysis. The water was removed during this measurement from the geopolymer using a slow heating process and continuous thermal effect recording. The thermal effect of removing the liquid from the concrete has been calculated using the software of the device (Table 4).

Additionally, the weight measurements of the dry and water-filled geopolymer were performed to determine the content of water in the pores. By analyzing the weight of water in the pores and the weight of the tested sample, open porosity can be calculated from the water density and its mass. The endothermic thermal effect of water evaporation and the energy absorbed by the solid geopolymer were recorded in parallel in the form of a peak in a DSC graph (Figure 4).

### 2.2. The Construction of the Measuring System

**Figure 2:**
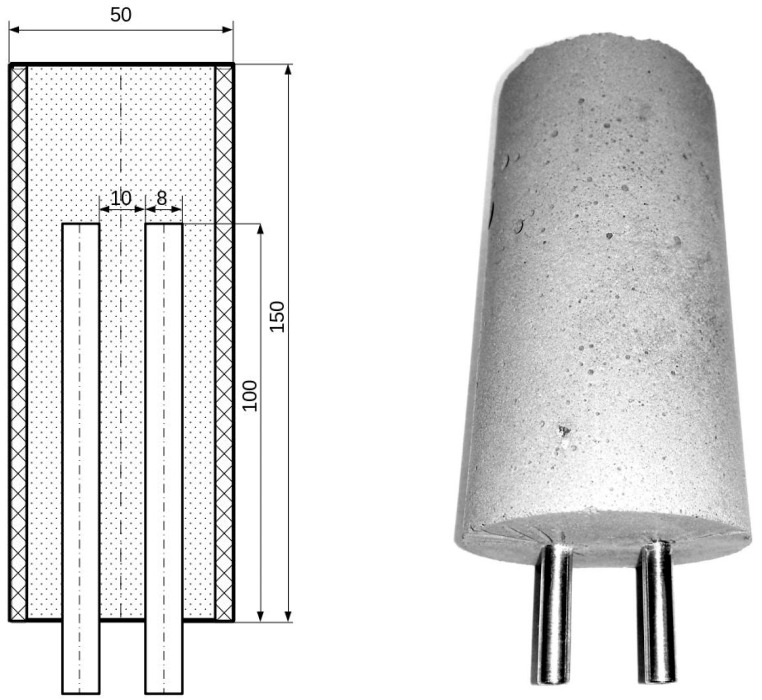
Measuring system and its photograph.

The geopolymer was prepared in polyvinyl chloride (PVC) tube, wherein two stainless steel electrodes were inserted. The construction of the measuring system and its photograph are shown in Figure 2. Immersing the electrodes in the material before the start of solidification ensured a good electrical connection between the electrodes and the geopolymer. This eliminated the impact of poor connection quality on the recorded curves.

## 3. Results

### 3.1. Mercury Porosity Measurements

The open porosity of the dry geopolymer was checked using the mercury porosimeter Anton Paar Poremaster 33. A sample geopolymer with a weight of 0.4022 g was used for porosity measuring. Measurements have been carried out in the range of mercury pressure from 0.1 MPa up to 400 MPa.

**Figure 3:**
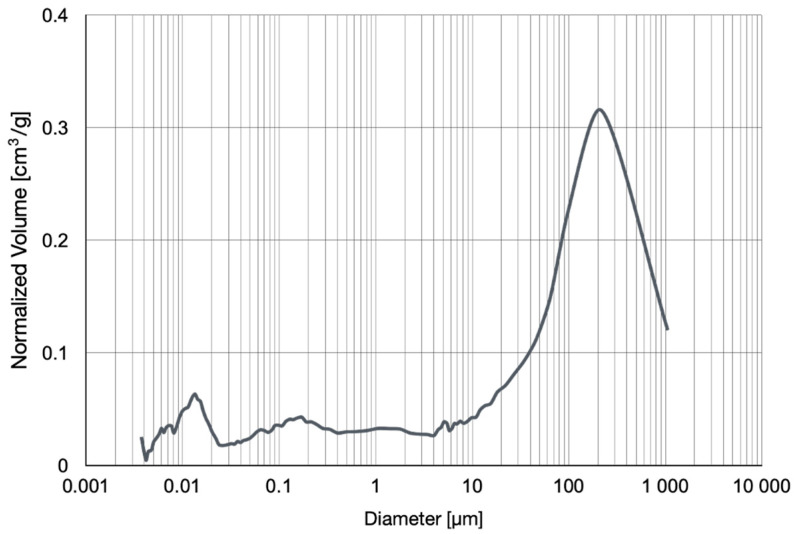
Mercury porosimeter measurements—normalized volume of pores’ diameter and their size.

Results are shown in Table 5 and Figure 3.

### 3.2. DSC Measurements

**Figure 4:**
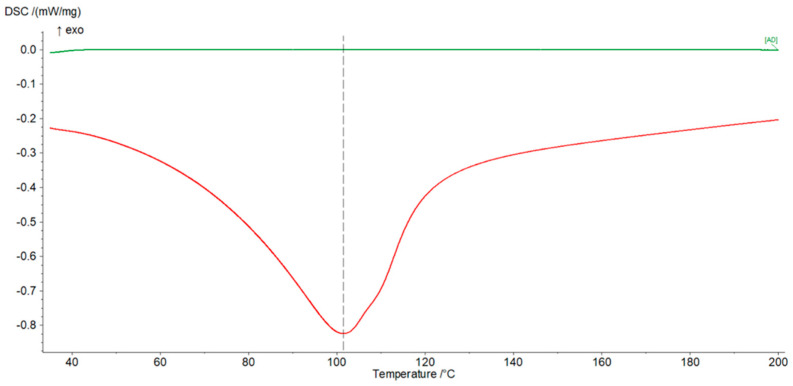
Differential scanning calorimetry graph. Endothermic effect of water evaporation—red curve. Thermal analysis of water-free geopolymer—green curve. The grey dotted line defines the maximum of the red curve and is 101.5 °C.

Measurements were carried out using the NETZSCH DSC 3500 device in the temperature range from 20 °C to 400 °C. The rate of heating and cooling of the sample was the same and amounted to 10 K/min. The first heating cycle (Figure 4—red curve) was aimed at evaporating the liquid from the previously soaked sample, and then, after cooling down, reheating was carried out (Figure 4—green curve) to check whether the observed thermal phenomena occur in the dry geopolymer in this temperature range.

After obtaining the specific volume of water which is the reciprocal of its density, the total volume of pores was calculated and shown in Table 6. The specific heat value of the geopolymer (dry material—Table 6) is not similar to the above-mentioned material, but its value corresponds well to the specific heat values of other similar materials such as concrete, natural stone or mortar.

### 3.3. Bode Curves and Related Graphs

**Figure 5:**
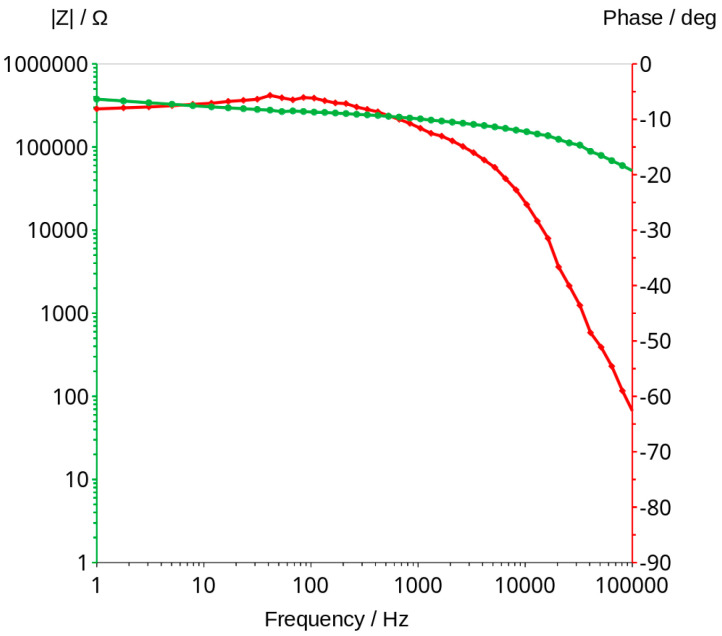
Bode graph of geopolymer after 80 h of drying in the temperature of 70 °C.

**Figure 6:**
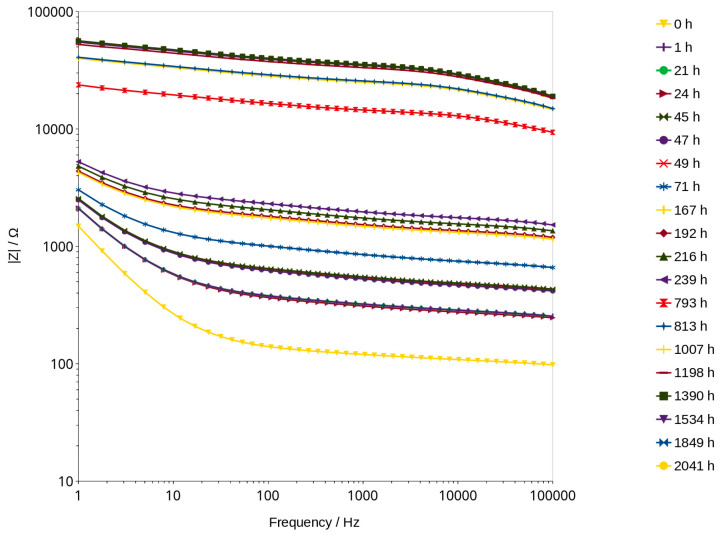
The impact of frequency on the impedance modulus as a function of time of drying.

Impedance measurements were carried out using Zahner IM6e electrochemical workstation. The measurements were taken in the frequency range between 1 Hz and 100 kHz with a sinusoidal voltage amplitude equal to 50 mV. Bode graphs, which usually consists of two curves, impedance module and phase shift vs. frequency (Figure 5), were presented in separate graphs for better a comparison of the changes taking place during the measurements (Figure 6).

**Figure 7:**
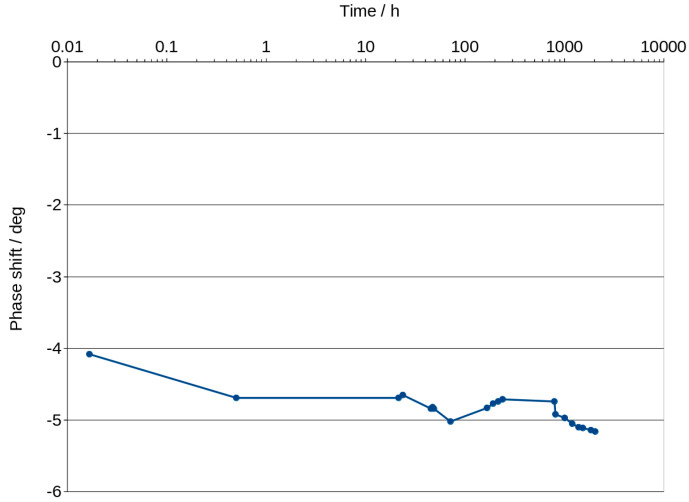
Changes in maximum value of phase shift vs. time.

**Figure 8:**
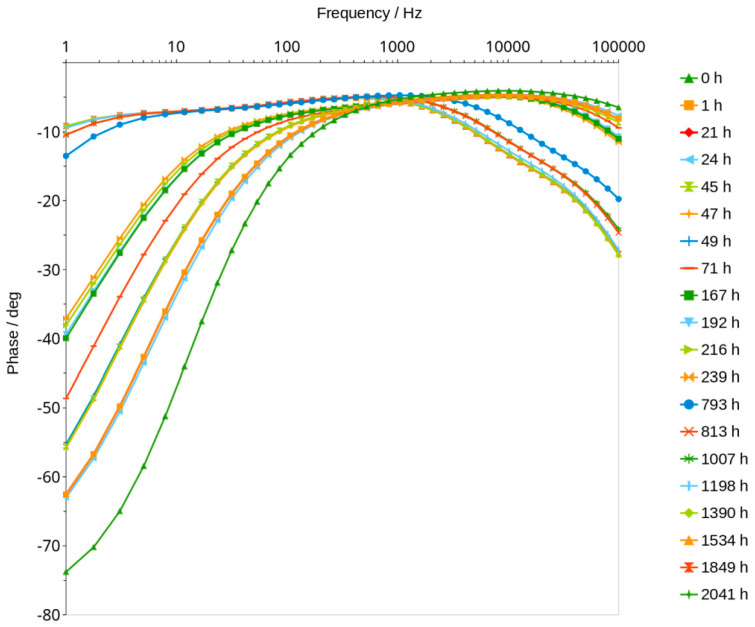
The impact of frequency on the phase shift angle as a function of time of drying.

**Figure 9:**
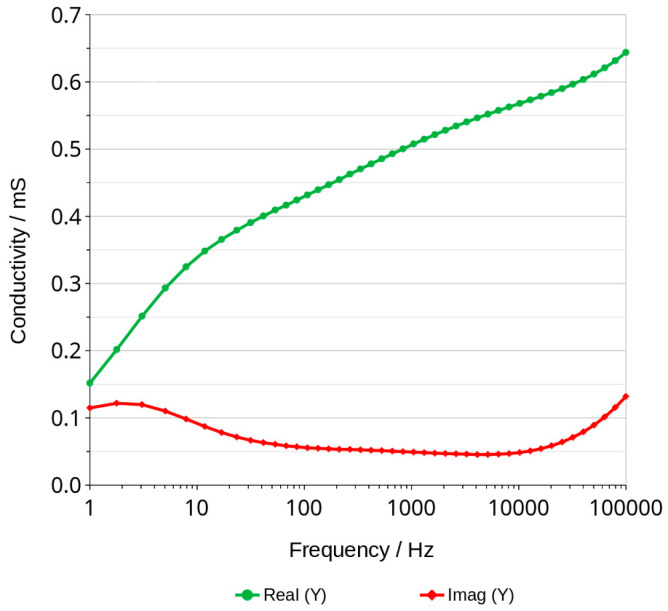
Example of conduction measurement of geopolymer after 239 h of drying.

**Figure 10:**
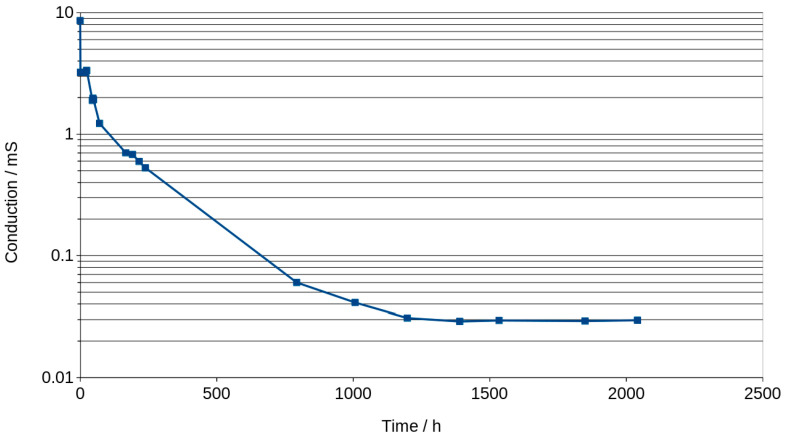
Conduction of material vs. time of drying calculated from Bode graph.

**Figure 11:**
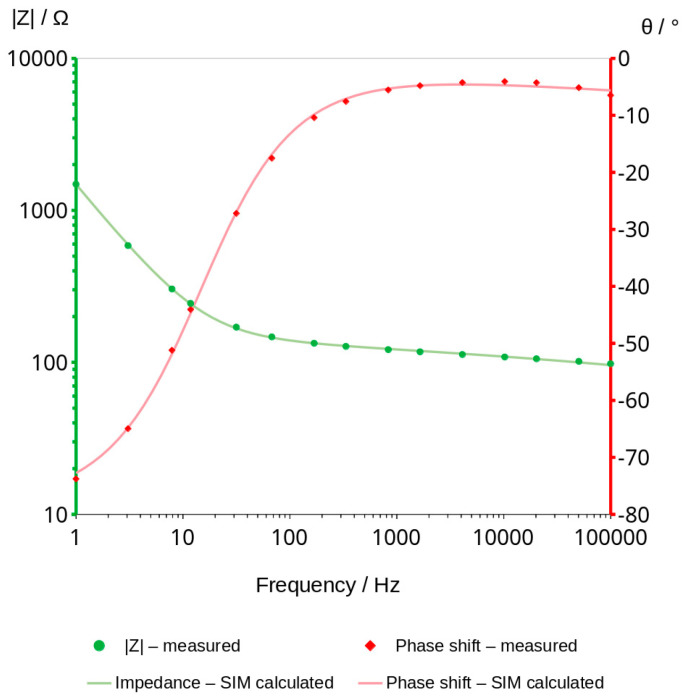
The example of fitting. The first measurement—0 h: fitting curves (lines) and measuring points (markers).

**Figure 12:**
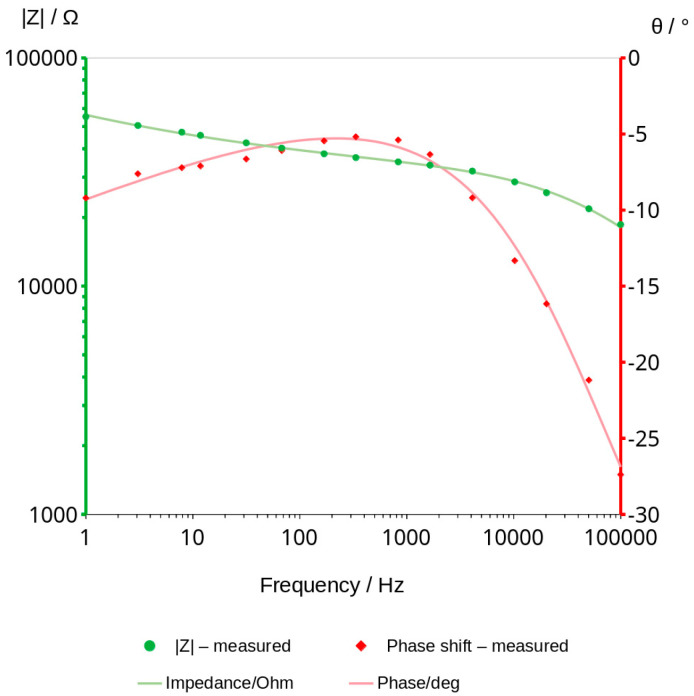
The example of fitting. The last measurement—2041 h: fitting curves (lines) and measuring points (markers).

Maximum phase shift angle oscillates around −5 deg (Figure 7). The mean calculated from all values of the graphs is equal to −4.89 deg. The exact value of conduction expressed in millisiemens (mS) cannot be obtained for this reason, but if we assume that −5 degrees is close to 0, we can calculate the conduction, but it will be slightly lower. Figure 9 shows an example of the changes in real and imaginary conduction measurements while frequency increases. Bode graphs indicate that maximum phase shifts angles occurs at frequencies of about 1000–2000 Hz (Figure 8, Figures 11 and 12). Looking at Figure 9, it can be seen that at around 2000 Hz frequency, value of the imaginary component of the impedance—Imag (Z)—is about 10 times smaller than the real component of the impedance value—Real (Z). Based on this observation and taking into account possible conduction error, its value was calculated directly from the Bode diagram of the module impedance |Z| obtained for 2000 Hz. Those values are shown in Figure 10. The conduction changes from about 9 mS for high water content in the material to 0.03 mS for the almost water-free material. The observed drops in values are very significant and make it possible to measure the conduction and approximate water content with a much simpler device than the one used in this study. Comparison measurements can easily be carried out using a 2000 Hz generator and an impedance meter.

### 3.4. Analysis of Graphs Using SIM Module

Electrochemical workstation Zahner IM6e is equipped with a software called simulation impedance module (SIM) that allows to simulate curves with known elements of from electrical circuits, like resistors and capacitors, and other elements characterized by special properties which can be applied in material investigations. Using this software and based on our experience, some schema that could well describe the phenomenon were proposed. After establishing the equivalent scheme, we started with more complex models, and then we removed, from the scheme, those elements that the software considered as not having a significant impact on the fitting of the measurement curves. A simplified model was chosen in which all the elements have a significant influence on the fit (Figure 11 and Figure 12).

**Figure 13:**
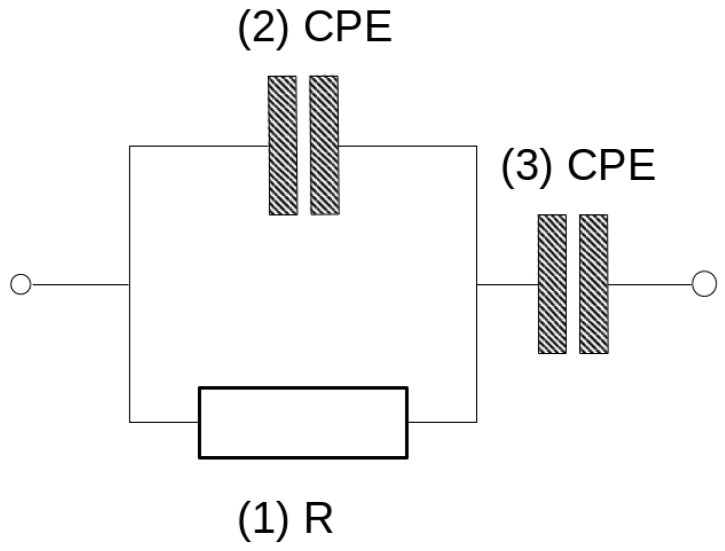
The equivalent scheme for good matching of measurement points—SIM model.

The model describes the mechanism of charge transfer and ensures a good fit of the points. The selected SIM model is shown in Figure 13.

**Figure 14:**
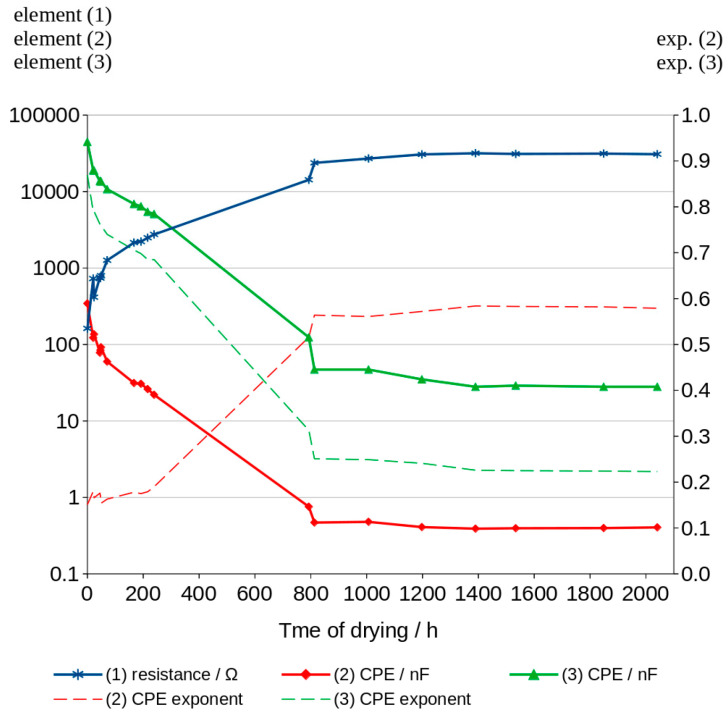
Changes in SIM model elements values.

The schema comprises three elements. The first one is resistor (1), the second is constant phase element CPE (2), connected in parallel to the resistor, and the third is the second CPE element (3), connected in series with the other two. The CPE element can be understood as a special type of capacitor in which capacitance coexists with its resistivity. Values of these elements are characterized by capacitance expressed typically in μF, nF or pF, and the exponent which can take values from 0 to 1. The exponent of CPE informs the main mechanism of charge transfer. The 0 value indicates resistive current flow and in that case CPE can be replaced in the model by a resistor. The value equal to 1 is characteristic of capacitor-type current flow. Values of calculated elements of the chosen model are shown in Table 7. The mean impedance module errors are not higher than 0.6% in all the obtained graphs and the phase error of calculated curves is less than 0.3%. Figure 14 shows the values of elements and the changes in them over time.

### 3.5. Nyquist Plots

Nyquist plots can be easily obtained in a relatively short time. Each measurement showed in this work took less than 2 min. The measurements were carried out in the same frequency range as for the Bode graphs.

## 4. Discussion

Mercury porosimeter measurements showed the total porosity of the investigated geopolymer as equal to 29.83% based on the intrusion of mercury into the material’s pores under high pressure. The mean value of pore diameter is 0.1269 μm. Pores smaller than 0.0095 μm make up 50% of the total number of pores. The volume of these pores is less than half of the total porosity (Table 5), so we found this that material consists of mainly higher than nano-size pores. The water, which was filled in the pores at a later stage, conducts electric charges and, as research has shown, is the only factor affecting conduction changes measurable with the methods used. Water-free geopolymer is non-conductive. The conduction mechanism changes with decreasing water content, which was illustrated by the changes in the values of the SIM model.

DSC measurements showed the total amount of water that filled the pores of the material. The absorbed water is removed during the slow heating of the geopolymer and is shown in the heating curve as an endothermic peak that appears near the boiling point of water—red line (Figure 4). Evaporation continues until the water is completely removed from the material. This process is slow due to the presence of small pores that hinder the movement of steam. As a result, the much wider than expected peak can also be observed. The recorded endothermic effect of the reaction is solely the result of evaporation of water from the sample. Thermal effects of other chemical reactions and phenomena were not found in the entire temperature range used. The recorded enthalpy of vaporization was used to approximate the porosity of the material (Table 6). The porosity of the investigated geopolymer achieved via DSC examination is about two times smaller than the total porosity measured via mercury porosimeter (Table 5). This is caused probably by the very high pressure of mercury applied in the device which can partially crash materials and open new pores. That was not the case in the material whose pores were filled with water. Reheating the sample (green line—Figure 4) showed no presence of water in the pores. The geopolymer was completely dry.

Impedance measurements can be used to monitor the changing water content in the geopolymers. They can probably be useful for testing other non-conductive porous materials stored in a humid environment. The accuracy of the method is sufficient to measure materials that contain very little or no water (Figure 5).

Analysis of water content can be carried out using a very low amplitude of voltage, which does not cause any change in the material. This method can be classified under non-destructive methods. The impedance modulus |Z| is generally frequency-dependent, but as shown in Figure 6, it shifts to higher values as the material dries. Curves shown in the mentioned figure are parallel to X axis at a frequency range of about 1000 to 2000 Hz. This indicates that conduction is close to resistance type in this range. It is not a fully resistive type because the impedance modulus |Z| slightly decreases with increasing frequency. The corresponding phase shift values in this range generally obtained the highest values. The maximum phase shift slightly decreases with time (Figure 8). Changes are measurable but the small difference of values does not have a significant influence on the mechanism of conduction. The mean value of about −4.89 deg is not far from zero compared to −90 deg which can be measured when conduction is represented only by capacitance (Figure 7). If it is assumed that the difference between −5 deg and 0 deg is irrelevant or small (Figure 8), it can be said that we do not observe the imaginary value of the impedance and conduction can be calculated based on the module of impedance (Figure 10). The value of the impedance modulus at the zero phase angle is equal to the resistance, and the conduction is the reciprocal of the resistance. Values of imaginary conduction Imag (Y) and real conduction Real (Y) of the completely dry material are shown in Figure 9. 

These values have a slight error because shape of the curves in the mentioned range indicates a small capacitance conduction. Nevertheless, such a value can be useful for a fast checking of the drying progress. 

Figure 8 shows the frequency of about 2000 Hz as a good choice for measuring conduction. Higher frequencies could be applied for much dried materials but looking at the phase shift angle related to those materials, it can be seen that the values are not much lower—about −5.1 deg compared to the mean value equal to −4.89 deg. This confirms that conduction with a small error can be measured for those materials using alternating current with a frequency of about 2000 Hz. 

The conduction of the geopolymer is shown in Figure 10. Values expressed in milisiemens (mS) start from 9 mS for very high content of water and decrease to 0.04 mS after 1500 h. This means that the material still contain small amounts of water which is in balance with the humidity of the environment. Totally dried geopolymer has no measurable values of conduction expressed in μS.

The simulation impedance module (SIM) was used to analyze the conduction mechanisms changing with the water content in the pores of the geopolymer. SIM analysis showed different mechanisms of charge transfer depending on the time of drying (Table 7; Figure 14). More resistive type of conduction can be observed in the first measurements (time of drying near to zero). The value of resistor (1) is the lowest and additionally, the value of the serially connected CPE (3) is highest in the first measurements. The corresponding value of its exponent is almost 0.9. This means that the charge transfers between electrodes occurs mainly through element 1 and element 3 due to the presence of a large number of conductive ions in the bigger pores. The conduction of ions in small pores can be expressed by the values of parallel connected CPE (2) in that range of time. When the geopolymer dries, resistance R (1) increases to higher values and CPE (3) decreases to lower values. Its exponent shows more resistive type of conduction. The values of CPE (2) decrease but conduction turns into more capacitive type. Values of CPE (2) exponent changed from 0.151 to 0.584.

**Figure 15:**
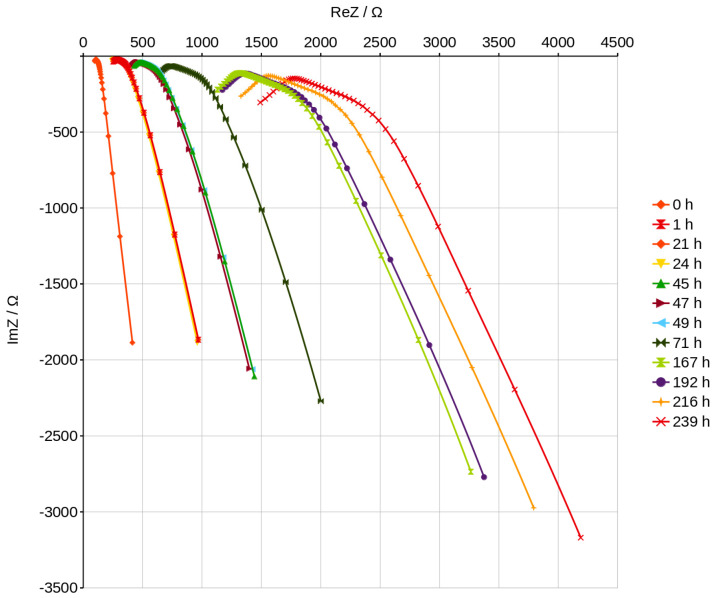
Nyquist plots for higher content of water—dependence on time.

**Figure 16:**
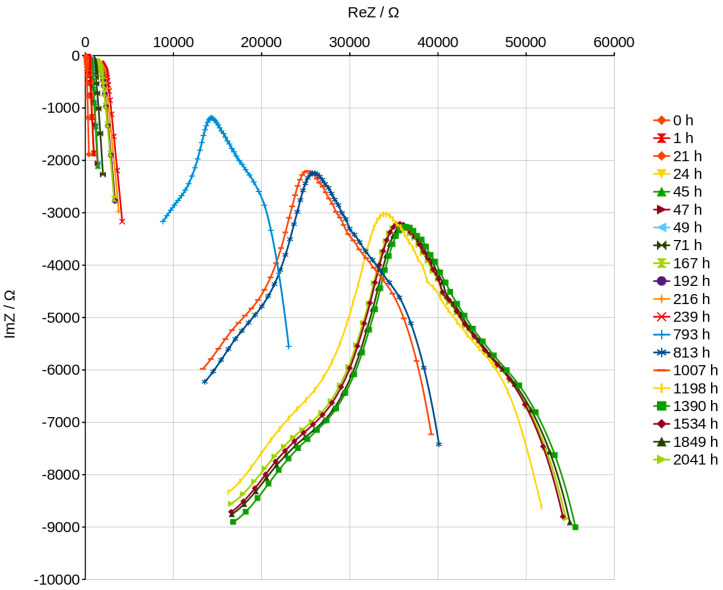
Nyquist plots for all measurements—dependence on time.

**Figure 17:**
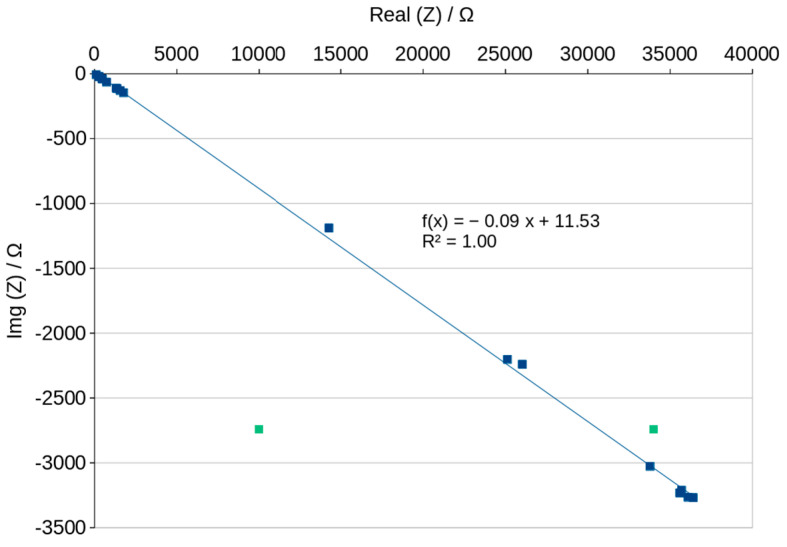
Nyquist plots peak position—dependence on time of drying.

The curves in the Nyquist graphs have a characteristic maximum value which can be noticed very precisely in the graphs plotted after about 70 h of slow drying (Figure 15). The analysis of the process of drying the geopolymer using these graphs can be very fast and easy to interpret because the graphs change significantly as drying progresses and the position changes in a characteristic way, as shown in Figure 15 and Figure 16. The peaks on the curves move to the higher ReZ and simultaneously to the lower ImZ values while the water content decreases. The dependence of the peak position on the content of water is shown in Figure 17 and can be described using a linear function. We noticed that the Nyquist curves have a characteristic peak (Figure 16), the location of which is shown in Figure 17 depending on the water content of the geopolymer. The position of this peak varies according to the linear function. If the laboratory has a device that allows to obtain a Nyqiust chart, it seems to be a simple and quick method of assessing the water content in the material and the progress in the drying process. If we determine the peak positions for two points, we can determine the function and estimate the time necessary to obtain the expected water content in the material. The equation will depend on the drying conditions, but the linear function reduces the number of necessary measurements. This present research is an attempt to check the possibility of measuring the wet-ability of porous geopolymer using impedance spectroscopy methods. The results have shown very good resolution of the method, thereby fulfilling the aim of our work. Further investigations could focus on measuring well-known water contents in the geopolymer and obtain the dependence of curves’ position. The applied method is very sensitive to the humidity of the environment in which the geopolymer is stored. It can be seen in Figure 16 that the peak position obtained after 1390 h of slow drying shows lower content of water than the peak obtained after 2041 h. The last measurements carried out between 1390 h and 2041 h shows the influence of the environment’s humidity. Sensitivity and resolution of the method could be probably useful for checking the wet-ability of building walls. Disadvantages of this method includes the necessity of preparing a special sample setup consisting of electrodes embedded in the material.

## 5. Conclusions

Geopolymers are porous materials because one could use liquid activators in the process of obtaining them. Removal of soluble materials from the solidified material result in its open porosity. The investigated concrete in this paper had a high porosity and a pore size distribution characteristic for geopolymers in which 8M activator is used. The proposed method of checking the water content in the pores during drying based on impedance measurements turned out to be useful. The dry geopolymer has a very high impedance module and the material can be considered as non-conductive. Small amount of water which could be caused by storing the concrete in normal humidity is measurable. The quickest and, in our opinion, the simplest method of comparing the water (or moisture) content of a sample is to measure the impedance at a frequency of 2000 Hz and calculate the conductivity using the value of the impedance modulus as mentioned above. At this frequency, most of the obtained Bode curves showed the value of the phase shift as closest to zero and calculated conduction with a small error. An advanced electrochemical workstation is not necessary for such a measurement. The correct measurement can be performed using an alternating current generator by setting the frequency to 20 kHz and limiting the voltage amplitude to 50 mV. EIS measurements carried out using electrochemical workstation open the possibility to track changes in the impedance occurring during the continuously changing frequency which is applied in this method. We propose that the closest real results can be obtained at the aforementioned frequency using this method. Comparison of the water content is also possible using the values of the elements of the equivalent scheme. The values of the elements in this diagram make it possible to determine the corresponding Bode and Nyquist curves. These values change significantly as the water content in the material decreases. When the water content in the material is high, conduction of charges is very easy. The flow of charges is mainly due to the resistor, and the high-capacity constant phase element CPE (3) connected in series with it, whose conduction is similar to that of a capacitor because the CPE exponent is close to 1. Conduction of the connected element in parallel with the resistor CPE (2) at a high water content is closer to that of a resistor than that of a capacitor and its value is low. When the water content in the pores decreases, the resistance R (1) increases rapidly and the values of the constant phase element decrease. The conduction character of CPE (3) begins to change to a more resistive one, while CPE (2) conducts in a mixed way, like both a resistor and a capacitor. Assuming that during drying, water evaporates more easily from larger pores, it can be stated that conductivity through small pores is mainly characterized by small CPE (2) values and its more capacitive character. The relationships between the values of the elements of the SIM scheme should depend on the distribution of porosity in the tested materials. However, it is necessary to carry out extended studies using materials with different porosity to confirm this. 

The most visible differences in measurements were obtained in the Nyquist graphs. All graphs showed a characteristic peak on the recorded curves, whose position linearly depends on water content in the pores. Small changes in the time of drying or ambient humidity fluctuations that increase or decrease evaporation can be recorded and easily seen in the graph without the need for additional calculations. The disadvantage of this method is the need to prepare a special measuring system in a repeatable manner so that it is possible to compare the materials produced, for example, with the use of a different activator. This should result in different concrete porosity as is reported in the literature. This has not yet been tested but materials with different porosity should have different conduction and this property can also be used to estimate porosity by measuring electrical quantities.

## Figures and Tables

**Table 1:** Classifications of fly ash [40].

Class of Fly Ash	N	F	C
SiO_2_ + Al_2_O_3_ + Fe_2_O_3_ (min. %)	70	70	50
SO_3_ (max. %)	4	5	5
Humidity (max. %)	3	3	3
Loss on ignition (max. %)	10	6	6

**Table 2:** The oxide composition of fly ash.

	SiO_2_	Al_2_O_3_	CaO	MgO	Na_2_O	K_2_O
[wt.%]	61.11 ± 1.20	26.45 ± 0.69	2.89 ± 0.29	0.98 ± 0.14	4.07 ± 0.26	4.50 ± 0.30

**Table 3:** Fly ash particle size distribution.

Particle Size [μm]	>160	100–160	71–100	63–71	56–63	<56
Contents [%]	0.3	3.2	11.9	9.9	15.4	59.3

**Table 4:** DSC result—red curve values.

T_pm_ [°C]	ΔH_m_ [J/g]	T_im_ [°C]	T_fm_ [°C]	DSC [mW/mg]
101.5	−184.4	70.9	123.0	−0.6236

T_pm_—temperature of peak maximum; T_im_—peak start temperature used for calculation; T_fm_—peak end temperature used for calculation; ΔH_m_—thermal effect of evaporation; power of evaporation.

**Table 5:** Mercury porosimeter measurements: statistical summary of pores.

Diameter of Pores	Mean	Median
	0.1269 μm	0.0095 μm
Volume of pores	0.401 cm^3^/g	0.227 cm^3^/g
Total intruded volume	0.455 cm^3^/g	
Total surface area	14.331 m^2^/g	
Mercury intrusion porosity	29.83%	

**Table 6:** Content of water in the geopolymer obtained via weight measurements and porosity-based DSC measurements.

	Weight [g]	Absorbed Energy by Mass [J/K]	Specific Heat * [J/g·K]
Wet material	0.0255	0.0465	1.8250
Dry material	0.0221	0.0323	1.4610
Water	0.0034	0.0143	4.1900
Porosity	13.36%		

*—Values of wet and dry geopolymer were obtained with dynamic changes in temperature and because of that they are not exact values of specific heat. The value shown for the distilled water was obtained from the tables highlighting physical quantities.

**Table 7:** Calculated values of SIM elements which fit the curves.

Time of Drying [h]	Element (1) [Ω]	Element (2) [nF]	Exp. of Element (2)	Element (3) [nF]	Exp. of Element (3)	|Z| Error [%]	Phase Shift Error [°]
0	161.7	342.50	0.151	44,480.0	0.869	0.3	0.1
21	427.1	122.30	0.180	18,860.0	0.790	0.5	0.2
24	424.8	136.00	0.166	18,860.0	0.720	0.5	0.2
45	758.0	78.10	0.176	13,660.0	0.762	0.5	0.2
47	738.3	84.00	0.169	13,750.0	0.760	0.5	0.2
49	793.2	92.18	0.154	13,540.0	0.758	0.5	0.2
71	1263.0	59.69	0.163	10,710.0	0.740	0.5	0.2
167	2148.0	31.32	0.178	6868.0	0.706	0.6	0.3
192	2216.0	30.59	0.175	6400.0	0.698	0.5	0.3
216	2487.0	26.07	0.179	5434.0	0.685	0.5	0.3
239	2747.0	21.94	0.190	5083.0	0.685	0.6	0.3
793	14,240.0	0.755	0.516	124.7	0.313	0.4	0.2
813	23,690.0	0.469	0.564	47.5	0.251	0.3	0.1
1007	23,020.0	0.479	0.561	47.4	0.249	0.3	0.1
1198	30,580.0	0.409	0.572	34.9	0.241	0.3	0.1
1390	31,670.0	0.390	0.584	28.4	0.226	0.3	0.1
1534	31,020.0	0.395	0.584	29.0	0.225	0.3	0.1
1849	31,320.0	0.397	0.583	28.4	0.225	0.3	0.1
2041	30,770.0	0.405	0.579	28.2	0.223	0.3	0.1

## Data Availability

Not applicable.
